# Novel Monte Carlo approach quantifies data assemblage utility and reveals power of integrating molecular and clinical information for cancer prognosis

**DOI:** 10.1038/srep15563

**Published:** 2015-10-27

**Authors:** Wim Verleyen, Simon P. Langdon, Dana Faratian, David J. Harrison, V. Anne Smith

**Affiliations:** 1 School of Biology, University of St Andrews, St Andrews, Fife, KY16 9TH, UK; 2 Division of Pathology, University of Edinburgh, Edinburgh, EH4 2XU, UK; 3 School of Medicine, University of St Andrews, St Andrews, Fife, KY16 9TF, UK

## Abstract

Current clinical practice in cancer stratifies patients based on tumour histology to determine prognosis. Molecular profiling has been hailed as the path towards personalised care, but molecular data are still typically analysed independently of known clinical information. Conventional clinical and histopathological data, if used, are added only to improve a molecular prediction, placing a high burden upon molecular data to be informative in isolation. Here, we develop a novel Monte Carlo analysis to evaluate the usefulness of data assemblages. We applied our analysis to varying assemblages of clinical data and molecular data in an ovarian cancer dataset, evaluating their ability to discriminate one-year progression-free survival (PFS) and three-year overall survival (OS). We found that Cox proportional hazard regression models based on both data types together provided greater discriminative ability than either alone. In particular, we show that proteomics data assemblages that alone were uninformative (p = 0.245 for PFS, p = 0.526 for OS) became informative when combined with clinical information (p = 0.022 for PFS, p = 0.048 for OS). Thus, concurrent analysis of clinical and molecular data enables exploitation of prognosis-relevant information that may not be accessible from independent analysis of these data types.

Most current clinical oncology practice stratifies patients based on tumour histology to inform prognosis. Molecular analyses are heralded as the solution for personalised medicine^1^, yet most such analyses view patients in segmented populations, either comparing molecular signatures across clinical and pathological categories^2^,^3^,^4^,^5^,^6^ or evaluating clinicopathological characteristics of clusters based upon molecular features^7^,^8^,^9^,^10^. This tends to underestimate the proven value of clinical and pathological information. When clinical and pathological information is used in combination with molecular analyses, it is typically in a *post-hoc* manner, that is, attempting to improve a molecular model with clinical information^11^. This places a high burden on molecular data, as it is required to be useful in isolation before the sequential addition of clinicopathological data. Here, we investigate a more integrative approach, using ovarian cancer as an example, where we analyse molecular and clinical data in concert. We take the point of view that molecular data should not *replace* traditional clinical pathology, but instead *add* to it.

We show the added value of molecular data in ovarian cancer, a disease with particularly poor prognosis: despite often initially good responses to chemotherapy, 65% die by 5 years^12^,^13^. There are no predictive biomarkers to direct specific treatment regimens^14^. Most patients undergo costly, neurotoxic platinum plus taxane therapy, though 20–30% do not respond. Alternative therapy with platinum only or, less commonly, lower toxicity agents can sometimes be equally effective^12^,^15^,^16^,^17^. Thus, personalising prognosis to enable better selection of these treatment options would be of great benefit in ovarian cancer.

We take advantage of the Edinburgh Ovarian Cancer Database^18^, a resource in which molecular data are available on samples with complete histopathology plus clinical outcomes. We develop a novel Monte Carlo approach to quantify the usefulness of different data assemblages and show that while proteomics data has low information content alone, selected informative proteomic features have high information content when viewed in the context of clinicopathological data.

## Results

**Figure 1:**
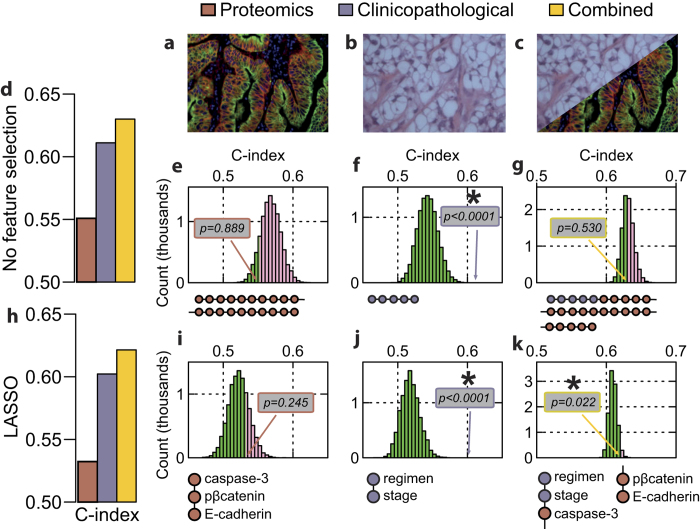
Added value of proteomics for predicting progression-free survival. (**a**–**c**) Example images representing proteomics, a fluorescence AQUA image (**a**) clinicopathology, a histological slice (**b**) and the combination (**c**). (**d**) C-index of Cox proportional hazards regression models for proteomics data only, clinicopathological data only, and combined proteomics and clinicopathological data. (**e**–**g**) Corresponding Monte Carlo (MC) analyses showing histograms of c-index from 10,000 randomised datasets; value of the actual analysis is highlighted and its p-value indicated (*-significant); histogram bars are coloured green below the actual value and pink above. (**h**–**k**) As for (**d**–**g**) after LASSO feature selection; selected features shown below MC histograms in order of decreasing hazard ratio. Note only proteomics data was randomised in (**g**) and (**k**).

We measured protein and phosphoprotein profiles of 339 clinically-annotated samples from the Edinburgh Ovarian Cancer Database (EOCD)^18^, including markers of proliferation, cell cycle, apoptosis, DNA damage response, estrogen signalling, and epithelial to mesenchymal (EMT) transition. We applied a Cox proportional hazards regression model (CPHR) for both progression-free survival (PFS) and overall survival (OS) to this proteomics data alone, clinicopathological data alone, and combined proteomics and clinicopathological data (Fig. 1a–c; measures detailed in Table 1; data available in Supplementary Data S1 and described in Supplementary Table S1). The combined models had higher concordance (c-index)^19^ than either data type alone (Fig. 1d for PFS; results for OS shown in Supplementary Fig. S1), indicating a greater discriminative ability; however, both the proteomics and combined models showed significant differences in cross-validation, suggesting potential overfitting (Supplementary Table S2).

**Figure 2:**
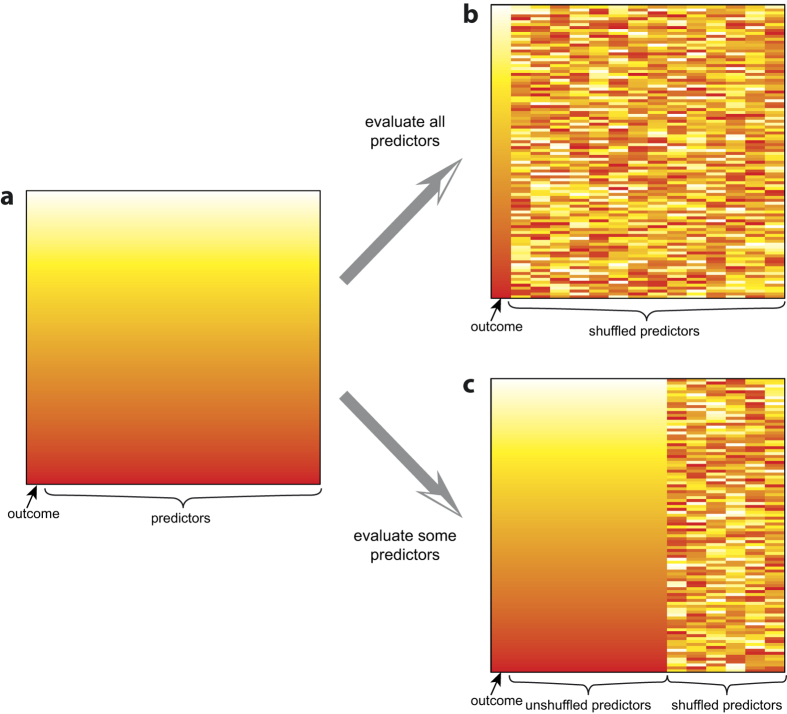
Shuffling methodology for novel Monte Carlo analysis. (**a**) Graphical representation of a dataset with patient outcome in the leftmost column and the remainder of the columns representing predictor variables; each row is coloured uniquely in a gradient to represent data from an individual patient for illustrative purposes. (**b**) For the Monte Carlo analysis, the values of each variable are shuffled, randomising that single variable with respect to patient outcome; this is carried out independently for each variable such that correspondence both between a variable and outcome, and among variables, is broken. Note this differs from standard Monte Carlo analyses, which would shuffle only patient outcome with respect to predictors, thus maintaining correspondence among variables. **(c**) The shuffling procedure can also be performed on a subset of variables, to evaluate only the added value of these variables.

We then developed a novel Monte Carlo (MC) method to assess the information content of variable assemblages, measuring their capacity to discriminate prognoses. We shuffled the values of the variables in question independently with respect to patient (Fig. 2), then built a CPHR, for each of 10,000 randomised datasets. A p-value was calculated as the proportion of randomised datasets with c-index equal to or above the actual model (one-tailed due to directional nature of the c-index). A high (non-significant) p-value indicates that the actual data discriminates prognoses little differently than does randomly assigned data, and thus the information content in that data assemblage is low; a low p-value indicates high information content and significant discriminative capacity.

The MC analysis revealed that the proteomic data alone had low information content (*P* = 0.889 for PFS, 0.617 for OS; Fig. 1e, Supplementary Fig. S1) while the clinicopathological data alone had high information content (*P* < 0.0001 for both PFS and OS; Fig. 1f, Supplementary Fig. S1). Since we were specifically interested in whether *adding* proteomics data to the already information-rich clinicopathological data was beneficial, we shuffled only the proteomics data in the combined model. This confirmed that the apparent increased discriminative ability of the combined model was an artefact (*P* = 0.530 for PFS, 0.117 for OS; Fig. 1g, Supplementary Fig. S1). This MC result held regardless of whether the c-index from the full model (as in Fig. 1) or a corrected c-index based on cross-validation was used (Supplementary Fig. S2).

We then applied LASSO feature selection^20^ to the data before building our CPHR models, to select only the most informative measures. Again, the combined models had greater discriminative ability than either individual model (Fig. 1h, Supplementary Fig. S1); this time, cross-validation showed no significant differences from the full models (Supplementary Table S2). However, the MC analysis revealed more detail: proteomics data alone still had low information content (*P* = 0.245 for PFS, 0.526 for OS; Fig. 1i, Supplementary Fig. S1) and clinicopathological high information content (*P* < 0.0001 for both PFS and OS; Fig. 1j, Supplementary Fig. S1), while the combined models now showed significantly increased discriminative capacity due to the added proteomics (*P* = 0.022 for PFS, 0.048 for OS; Fig. 1k, Supplementary Fig. S1). Again, the MC result also held if a corrected c-index based on cross validation was used (Supplementary Fig. S2); thus, the significant increase was not due to overfitting in the context of the full model. Because only the proteomics data were shuffled in the combined model, the results in Fig. 1i and Fig. 1k are directly comparable: proteomics data, which alone had low information content, showed added value when used alongside clinicopathological information.

This was not true for the entire proteomics profile, however (Fig. 1e compared to Fig. 1g); thus, only carefully selected molecular measures can significantly increase discriminative ability above that provided by clinicopathological information. Figure 1i–k and Supplementary Fig. S1 show the features selected for PFS and OS, respectively.

## Discussion

Our work demonstrates the power of concurrent integration of traditional histopathology plus newer molecular measures to create something greater than either alone. Using proteomic profiles of samples with complete clinicopathological data, we have shown how incorporating molecular alongside clinicopathological data improves survival analyses. In doing so, we have developed a novel Monte Carlo analysis to quantify the usefulness of data assemblages.

Machine learning methodologies in molecular analyses of cancer have been criticised for overfitting problems^21^, and we directly address this problem with our Monte Carlo analysis. We reveal data assemblages with low information content yet high performance, whose performance must then be due to overfitting. Where 10-fold cross validation of the c-index suggested overfitting issues, our MC analysis agreed, showing low information content for both proteomics alone and combined datasets with no feature selection. However, our MC analysis provided further information where cross-validation showed no significant differences, revealing low information content in selected proteomics features alone. Only when these proteomics features were combined with selected clinical features did they prove to be informative.

We found that feature selection before survival analysis is key to producing sensible information out of the molecular data. Using all available proteomic measures in addition to clinicopathological data at first appears to increase the discriminatory ability of survival analysis, but this is in fact due to overfitting. However, if feature selection is first applied, the addition of proteomic to clinicopathological data significantly increases the discriminatory ability of our CPHR model. The measures selected provide insights into the biology of ovarian cancer. E-cadherin is related to cell adhesion, and its loss has been reported to be associated with poor survival^22^,^23^,^24^. Caspase-3 perhaps indicates benefits of propensity to apoptosis, and has been associated with more favourable patient outcomes^25^,^26^. pH2AX is a marker of DNA damage repair, while expression of the Wilms’ tumour 1 (WT1) gene has been associated with poor prognosis in ovarian cancer^27^,^28^. In contrast, nuclear beta-catenin expression has been associated with favourable outcomes in this disease^29^,^30^,^31^.

There is merit in further examination of the data, because the details reveal important features. Comparing Fig. 1d,h reveals that the CPHR models that contain all the proteomic data are more discriminatory (higher c-index) than those with only selected proteomic measures; however, we know this is due to overfitting from the MC analysis (Fig. 1g). Yet even the selected proteomics measures alone have poor discrimination (c-index close to 0.5) and non-significant MC p-values (Fig. 1i), indicating low information content. Only when these selected proteomics measures are combined with clinicopathological measures do we see improvement in the c-index and significant information content revealed by MC analysis (Fig. 1k). In particular, this MC analysis is directly comparable to that with just proteomics: since only the proteomics variables are shuffled, only the information content of these proteomics measures are revealed. Thus, the information content of the proteomics differs depending on the context. The proteomic data, which alone was uninformative, added value when used alongside clinicopathological information.

The above shows the power of our MC approach for assessing data assemblages. The information content of a data set can be assessed as a whole by shuffling all variables; alternatively, shuffling only those additional variables assesses the benefit of adding specific measurements to an already useful group of features. Thus, we present a method of quantifying usefulness of measures when direct success of a model may be less meaningful due to overfitting concerns. This quantification methodology could be applied to evaluate the discriminative ability of features used to assess patient outcome in many diseases, a necessary step for personalised medicine.

Our work demonstrates the path towards a systems pathology approach for personalised medicine. We move beyond sequential application of clinicopathological and molecular data to stratify groups or to refine models. We analyse proteomics data in concert with traditional histology and clinical measures, enabling better discrimination than either alone. This was true even though the proteomics data was uninformative alone, a stage at which many such molecular studies might otherwise be abandoned. Our Monte Carlo-based assessment of information content can quantify the added value of new data, thus both enabling the identification of beneficial variable additions and avoiding overfitting. Our results generalise to other diseases where long-established pathological analyses already produce valuable information that should not be ignored.

## Methods

### Study Population

Formalin-fixed, paraffin-embedded ovarian tumour samples were obtained from the Edinburgh Ovarian Cancer Database (EOCD) as previously described^8^,^18^. The data set consisted of 339 samples, which form a subset of those analysed in Faratian *et al.*^8^. This research was approved by the Lothian Research Ethics Committee (08/S1101/41).

### Clinicopathological Measures

Samples in the EOCD were annotated with clinicopathological information which were divided into “input” measures—those relating to patient, disease, and treatment characteristics—and “output” measures—those relating to survival. A summary of the clinicopathological measures is shown in Table 1; data are available in Supplementary Data S1 and described in Supplementary Table S1. The output measure of progression-free survival (PFS) represents the number of days between the start of treatment and the first signs of cancer recurrence; overall survival (OS) represents the number of days between the first histological diagnosis and the day of death. Both survival measures were right-censored.

### Proteomic Measures

Proteins and subcellular location measured are shown in Table 1. Protein and phosphoprotein levels were obtained by automated quantitative immunofluorescence using carefully validated antibodies as previously described^8^. Briefly, tissue microarrays were constructed using triplicate samples from each tumour. Immunofluorescence detection of phosphoprotein and other targets was performed using methods previously described^8^,^32^; antibodies and conditions used are shown in Supplementary Table S3. Pan-cytokeratin antibody was used to identify infiltrating tumour cells, DAPI counterstain to identify nuclei, and Cy-5-tyramide detection of target for compartmentalised (tissue and subcellular) analysis of tissue sections. Monochromatic images of each TMA core were captured at x20 objective using an Olympus AX-51 epifluorescence microscope, and high-resolution digital images analysed by the AQUAnalysis^TM^ software. If the epithelium comprised <5% of total core area, the core was excluded from analysis. Protein and phosphoprotein expression was quantified by calculating the Cy5 fluorescence signal intensity on a scale of 0–255 within each image pixel, and the AQUA score generated by dividing the sum of Cy5 signal within the epithelium by the area of the cytoplasm or nucleus for cytoplasmic or nuclear measurements, respectively. AQUA scores were averaged from triplicate cores and mean values obtained.

### Survival Analysis

Cox proportional hazards regression (CHPR) was applied to clinicopathological inputs and proteomic measures, using the cph function in the R package rms (Breslow method; x and y set to ‘TRUE’ for use in cross-validation, below), to predict both PFS and OS. Models without feature selection were full multivariate models using all measures in Table 1; models using LASSO feature selection were multivariate models including those features as noted in Fig. 1 and Supplementary Figure S1. Validity of the proportional hazards assumption was assessed using visual inspection of plots from the R functions survplot and cox.zph, and examination of statistics of Schoenfeld residuals. Coefficients with 95% confidence intervals and associated Schoenfeld residual statistics for all models are presented in Supplementary Table S4. CPHR models were assessed using the concordance index (c-index)^19^, available from the R function validate. The c-index represents the probability that, for two randomly chosen patients, the model correctly orders the patients in their outcome measure (here PFS and OS). Ten-fold cross-validation was performed computing the c-index for each resample (dxy = ‘TRUE’), and repeated 100 times to provide average performance in cross-validation.

### Feature Selection

Feature selection was performed using the least absolute shrinkage and selection operator (LASSO)^20^ to identify the most informative features for OS and PFS. LASSO was applied using functions optL1 and profL1 in the R package penalized (and verified with glmnet); the sparsity parameter (λ) was obtained by a likelihood cross-validation with settings: 10-folds and the sparsity parameter lies in the interval: 0.001 < λ < 50.

### Monte Carlo Analysis

We developed a novel Monte Carlo analysis to evaluate information content of any variable assemblage. Figure 2 describes the shuffling methodology graphically: each variable is shuffled independently of all others and of patient outcome; all variables or a subset can be shuffled to analyse the information content of the entire assemblage or a particular group, respectively. This methodology can be applied with any analysis method that provides a scalar performance measure; we applied it to CHPR models evaluated via the c-index (see Results). R code to perform our Monte Carlo analysis for CHPR models is provided as Supplementary Data S2; an example vignette applying it to our data is available as Supplementary Note S1.

## Additional Information

**How to cite this article**: Verleyen, W. *et al.* Novel Monte Carlo approach quantifies data assemblage utility and reveals power of integrating molecular and clinical information for cancer prognosis. *Sci. Rep.* **5**, 15563; doi: 10.1038/srep15563 (2015).

## Supplementary Material

Supplementary Information

Supplementary Data S1

Supplementary Data S2

## Figures and Tables

**Table 1:** Clinicopathological and proteomic measures.

*Clinicopathological*		*Proteomic*		
*Measure*	*Values*	*Protein/phosphoprotein*	*Measured in*	
			*Nucleus*	*Cytoplasm*
***inputs***		pERK	x	
age	continuous (days)	pβCatenin	x	
	stratified < >50 years	pSTAT3 (Ser727)	x	
histopathology	papillary serous	pSTAT3 (Ser705)	x	
	clear cell	pNFkB	x	
	endometrioid	pRB	x	
	mixed histology	pH2AX	x	
	mucinous	pBRCA1	x	
	adenocarcinoma	p-p53	x	
stage	stage 1	Ki67	x	
	stage 2	phosphohistone H3 (pHH3)	x	
	stage 3	cleaved caspase-3	x	
	stage 4	WT1	x	
regimen	platinum	Snail		x
	platinum + taxane	Slug		x
***outputs***		E-cadherin		x
progression-free survival	continuous (days)	estrogen receptor-β 1 (ERβ1)	x	x
overall survival	continuous (days)	estrogen receptor-β 2 (ERβ2)	x	x
