# Supplementary material for: Novel Monte Carlo approach quantifies data assemblage utility and reveals power of integrating molecular and clinical information for cancer prognosis
Source: Sci Rep. 2015 Oct 27;5:15563. doi: 10.1038/srep15563 (PMC4622081; doi:10.1038/srep15563)
Supplement: Supplementary Information [file srep15563-s1.doc]

# Supplementary Information

# Novel Monte Carlo approach quantifies data assemblage utility and reveals power of integrating molecular and clinical information for cancer prognosis

Wim Verleyen^1†^, Simon P. Langdon^2^, Dana Faratian^2^, David J. Harrison^3^, V. Anne Smith^1*^

^1^School of Biology, University of St Andrews, St Andrews, Fife, KY16 9TH, UK.

^2^Division of Pathology, University of Edinburgh, Edinburgh, EH4 2XU, UK.

^3^School of Medicine, University of St Andrews, St Andrews, Fife, KY16 9TF, UK.

^†^Current address: Stanley Institute for Cognitive Genomics, Cold Spring Harbor Laboratory, 500 Sunnyside Boulevard, Woodbury, NY 11797, USA.

^*^corresponding author: anne.smith@st-andrews.ac.uk

## Figures

**Supplementary Figure S1. Added value of proteomics for predicting overall survival.** **(a-c)** Example images representing proteomics data, a fluorescence AQUA image **(a)**, clinicopathological data, a histological slice **(b)**, and the combination **(c)**. **(d)** C-index of Cox proportional hazards regression (CPHR) models for proteomics data only, clinicopathological data only, and combined proteomics and clinicopathological data. **(e-g)** Corresponding Monte Carlo (MC) analyses showing histograms of distribution of c-index values from 10,000 randomised datasets; value of the actual analysis is highlighted and its p-value indicated (*-significant); histogram bars are coloured green below the actual value and pink above. **(h-k)** As for (d-g) after LASSO feature selection; selected features shown below MC histograms in order of decreasing hazard ratio. Note only proteomics data was randomised in **(g)** and **(k)**.

**Supplementary Figure S2. Monte Carlo analysis using corrected c-index based on cross-validation.** Corrected c-index was calculated as mean over the corrected estimate from 100 repetitions of 10-fold cross-validation, for both the actual analysis and for each of 10,000 randomised datasets. Histograms of distribution from randomised datasets are shown with value of the actual analysis is highlighted and its p-value indicated (*-significant); histogram bars are coloured green below the actual value and pink above. Note that only the proteomics data was shuffled in the combined models.

## Tables

**Supplementary Table S1.** Clinical and pathological features of dataset.

| *Clinical* | |  | *Pathological* | |
| --- | --- | --- | --- | --- |
| ***Age*** | |  | ***Histology*** (No. each / %) | |
| median | 61.19 yrs |  | serous | 209 / 62% |
| range | 27.35 - 86.06 yrs |  | clear cell | 14 / 4% |
| No. < / > 50 yrs | 51 / 288 |  | endometriod | 58 / 17% |
| ***Progression-free survival*** | |  | mixed histologies | 42 / 12% |
| median | 357 days |  | mucinous | 7 / 2% |
| range | 2 days - 9.15 yrs |  | adenocarcinoma | 9 / 3% |
| No. ≤ / > 1 yr | 173 / 166 |  | ***Stage*** (No. each / %) | |
| ***Overall survival*** | |  | stage 1 | 21 / 6% |
| median | 2.94 yrs |  | stage 2 | 30 / 9% |
| range | 37 days - 17.42 yrs |  | stage 3 | 222 / 65% |
| No. ≤ / > 3 yrs | 212 / 127 |  | stage 4 | 66 / 19% |
|  |  |  | ***Regimen*** (No. each / %) | |
|  |  |  | platinum | 213 / 63% |
|  |  |  | platinum + taxane | 126 / 37% |

**Supplementary Table S2. Statistics from ten-fold cross-validation of Cox proportional hazards models.**

| *C-index* | | *100 repetitions of 10-fold CV (Mean±SE)* | | *Test compared to Full* | | |
| --- | --- | --- | --- | --- | --- | --- |
|  |  |  |  | *P-value* | *95% CI* | |
| *Model* | *Full* | *Train* | *Test* | *Mean* | *Lower* | *Upper* |
| ***No feature selection*** | | | | | | |
| PFS-Proteomics | 0.551 | 0.557±9.6E-05 | 0.474±1.0E-03 | 0.004* | 0.002 | 0.007 |
| PFS-Clinicopathological | 0.611 | 0.612±8.2E-05 | 0.591±6.9E-04 | 0.328 | 0.291 | 0.365 |
| PFS-Combined | 0.630 | 0.633±5.4E-05 | 0.572±7.5E-04 | 0.020* | 0.016 | 0.024 |
| OS-Proteomics | 0.567 | 0.570±7.7E-05 | 0.495±1.1E-03 | 0.007* | 0.005 | 0.009 |
| OS-Clinicopathological | 0.626 | 0.625±4.7E-05 | 0.606±6.1E-04 | 0.337 | 0.302 | 0.372 |
| OS-Combined | 0.647 | 0.650±5.3E-05 | 0.594±8.4E-04 | 0.027* | 0.018 | 0.035 |
| ***LASSO feature selection*** | | | | | | |
| PFS-Proteomics | 0.532 | 0.533±4.6E-05 | 0.522±8.0E-04 | 0.583 | 0.536 | 0.629 |
| PFS-Clinicopathological | 0.602 | 0.602±4.0E-05 | 0.601±4.9E-04 | 0.822 | 0.795 | 0.848 |
| PFS-Combined | 0.621 | 0.621±2.6E-05 | 0.612±6.2E-04 | 0.620 | 0.581 | 0.660 |
| OS-Proteomics | 0.531 | 0.533±5.8E-05 | 0.514±7.2E-04 | 0.398 | 0.356 | 0.439 |
| OS-Clinicopathological | 0.605 | 0.605±1.9E-05 | 0.604±5.5E-04 | 0.813 | 0.782 | 0.844 |
| OS-Combined | 0.624 | 0.625±3.1E-05 | 0.612±7.0E-04 | 0.507 | 0.460 | 0.554 |

*-C-index of full model significantly different from cross-validation in one-sample t-test

**Supplementary Table S3.** Antibodies and conditions used for automated quantitative immunofluorescence.

| *Protein/phosphoprotein* | *Antibody supplier* | *Dilution (AQUA)* | *Antigen retrieval* |
| --- | --- | --- | --- |
| pERK (Thr202/204) | CST (#9101) | 1:25 | Sodium citrate pH6.0 |
| pβCatenin (Ser33/37/Thr41) | CST (#9561) | 1:25 | Sodium citrate pH6.0 |
| pSTAT3 (Ser727) | CST (#9134) | 1:100 | Sodium citrate pH6.0 |
| pSTAT3 (Ser705) | CST (#9145) | 1:25 | Sodium citrate pH6.0 |
| pNFkB (Ser276) | CST (#3037) | 1:25 | Sodium citrate pH6.0 |
| pRB (Ser807/811) | CST (#9308) | 1:50 | Sodium citrate pH6.0 |
| pH2AX (Ser139) | CST (#9718) | 1:50 | Sodium citrate pH6.0 |
| pBRCA1 (Ser1524) | CST (#9009) | 1:25 | Sodium citrate pH6.0 |
| p-p53 (Ser15) | CST (#9286) | 1:100 | Sodium citrate pH6.0 |
| Ki67 | DAKO (M7240) | 1:50 | Tris-EDTA pH9.0 |
| pHH3 | CST (#9701) | 1:100 | Tris-EDTA pH9.0 |
| caspase-3 | CST (#9661) | 1:200 | Sodium citrate pH6.0 |
| WT1 | Genetex | 1:100 | Sodium citrate pH6.0 |
| Snail | Abcam | 1:800 | Sodium citrate pH6.0 |
| Slug | Lifespan Bio | 1:1000 | Sodium citrate pH6.0 |
| E-cadherin | BD Sciences | 1:1500 | Sodium citrate pH6.0 |
| ERß1 | Serotec(MCA1947T) | 1:10 | Sodium citrate pH6.0 |
| ERß2 | Serotec(MCA2279ST) | 1:400 | Sodium citrate pH6.0 |

**Supplementary Table S4. Coefficients for Cox proportional hazards regression.** Coefficient values for all variables in each Cox proportional hazards regression model are shown, along with lower and upper 95% confidence intervals and statistics of the Schoenfeld residuals for that variable (the correlation rho, the associated chi-square statistic and its p-value).

|  |  | *95% confidence interval* | | *Schoenfeld residual statistics* | | |
| --- | --- | --- | --- | --- | --- | --- |
| *variable* | *coefficient* | *lower* | *upper* | *rho* | *chi-square* | *P* |
| ***Progression-free survival, no feature selection*** | | | |  |  |  |
| ***Proteomics*** | | | |  |  |  |
| pERK_nuclei | -4.29E-04 | -1.67E-03 | 8.11E-04 | 0.02 | 0.21 | 0.649 |
| pSTAT3_727_nuclei | 6.70E-05 | -1.24E-03 | 1.38E-03 | -0.10 | 3.55 | 0.060 |
| pSTAT3_705_nuclei | -4.01E-04 | -1.91E-03 | 1.11E-03 | 0.06 | 1.66 | 0.198 |
| pNFkB_nuclei | -8.96E-04 | -4.09E-03 | 2.29E-03 | 0.00 | 0.00 | 0.947 |
| pBRCA1_nuclei | 1.85E-04 | -5.37E-03 | 5.74E-03 | -0.08 | 2.67 | 0.102 |
| pH2AX_nuclei | 9.32E-06 | -1.45E-03 | 1.47E-03 | -0.04 | 0.53 | 0.467 |
| pRB_nuclei | 3.50E-04 | -5.40E-04 | 1.24E-03 | 0.06 | 1.13 | 0.288 |
| pP53_nuclei | -1.91E-03 | -4.46E-03 | 6.41E-04 | -0.06 | 1.15 | 0.284 |
| Ki67_nuclei | -1.51E-04 | -5.07E-04 | 2.05E-04 | -0.03 | 0.47 | 0.492 |
| pHH3_nuclei | 5.85E-03 | -5.91E-03 | 1.76E-02 | 0.06 | 1.65 | 0.198 |
| caspase3_nuclei | 1.49E-02 | -5.29E-03 | 3.51E-02 | -0.02 | 0.24 | 0.627 |
| pBetaCatenin_nuclei | 1.85E-03 | -1.93E-03 | 5.64E-03 | 0.03 | 0.33 | 0.566 |
| Erbeta1_nuclei | -6.76E-04 | -2.62E-03 | 1.26E-03 | 0.01 | 0.05 | 0.827 |
| Erbeta1_cytoplasmic | 1.27E-03 | -3.18E-03 | 5.72E-03 | -0.02 | 0.15 | 0.698 |
| Erbeta2_nuclei | 4.72E-03 | -2.09E-03 | 1.15E-02 | 0.00 | 0.00 | 0.960 |
| Erbeta2_cytoplasmic | -8.80E-03 | -1.95E-02 | 1.94E-03 | 0.06 | 1.58 | 0.208 |
| WT1_nuclei | 2.29E-05 | -2.95E-05 | 7.53E-05 | 0.05 | 1.07 | 0.302 |
| Ecadherin_cytoplasm | -3.47E-05 | -9.58E-05 | 2.64E-05 | 0.01 | 0.02 | 0.892 |
| Slug_cytoplasm | 1.91E-05 | -6.84E-05 | 1.07E-04 | 0.03 | 0.34 | 0.558 |
| Snail_nuclei | -2.54E-05 | -9.33E-05 | 4.25E-05 | -0.01 | 0.07 | 0.787 |
| ***Clinicopathological*** | | | |  |  |  |
| Age | 1.05E-05 | -2.87E-05 | 4.96E-05 | 0.11 | 4.26 | 0.039 |
| AgeStratified | -1.92E-01 | -5.97E-01 | 2.14E-01 | -0.05 | 0.67 | 0.412 |
| HistologicalType=clear_cell | -2.88E-01 | -1.13E+00 | 5.56E-01 | 0.05 | 0.83 | 0.363 |
| HistologicalType=endometrioid | -3.53E-01 | -1.06E+00 | 3.59E-01 | 0.02 | 0.08 | 0.780 |
| HistologicalType=mixed_histology | -2.05E-01 | -9.43E-01 | 5.34E-01 | -0.04 | 0.48 | 0.487 |
| HistologicalType=mucinous | -1.02E-01 | -1.10E+00 | 8.94E-01 | -0.02 | 0.11 | 0.741 |
| HistologicalType=papillary_serous | -2.20E-01 | -8.98E-01 | 4.57E-01 | 0.00 | 0.00 | 0.966 |
| Stage | 4.26E-01 | 2.69E-01 | 5.82E-01 | 0.03 | 0.23 | 0.634 |
| Regimen=platinum+taxane | -3.40E-01 | -5.81E-01 | -9.93E-02 | 0.18 | 11.44 | 0.001 |
| ***Combined*** | | | |  |  |  |
| Age | 1.69E-05 | -2.51E-05 | 5.90E-05 | 0.14 | 7.38 | 0.007 |
| AgeStratified | -2.11E-01 | -6.42E-01 | 2.19E-01 | -0.06 | 1.47 | 0.226 |
| HistologicalType=clear_cell | -7.32E-01 | -1.72E+00 | 2.57E-01 | 0.03 | 0.48 | 0.489 |
| HistologicalType=endometrioid | -3.45E-01 | -1.12E+00 | 4.28E-01 | 0.00 | 0.00 | 0.955 |
| HistologicalType=mixed_histology | -1.83E-01 | -9.78E-01 | 6.11E-01 | -0.05 | 1.02 | 0.313 |
| HistologicalType=mucinous | 2.89E-02 | -1.05E+00 | 1.10E+00 | -0.01 | 0.07 | 0.788 |
| HistologicalType=papillary_serous | -1.74E-01 | -9.04E-01 | 5.56E-01 | -0.02 | 0.16 | 0.693 |
| Stage | 5.05E-01 | 3.38E-01 | 6.73E-01 | 0.02 | 0.09 | 0.762 |
| Regimen=platinum+taxane | -3.27E-01 | -5.85E-01 | -6.86E-02 | 0.20 | 14.36 | 0.0002 |
| pERK_nuclei | -8.41E-04 | -2.11E-03 | 4.29E-04 | 0.02 | 0.10 | 0.754 |
| pSTAT3_727_nuclei | 6.10E-04 | -7.83E-04 | 2.00E-03 | -0.11 | 4.31 | 0.038 |
| pSTAT3_705_nuclei | -9.18E-04 | -2.51E-03 | 6.79E-04 | 0.07 | 1.94 | 0.164 |
| pNFkB_nuclei | -1.81E-03 | -5.12E-03 | 1.50E-03 | 0.01 | 0.06 | 0.811 |
| pBRCA1_nuclei | -1.08E-03 | -6.60E-03 | 4.44E-03 | -0.07 | 2.36 | 0.125 |
| pH2AX_nuclei | 2.69E-04 | -1.49E-03 | 2.02E-03 | -0.05 | 1.01 | 0.315 |
| pRB_nuclei | 4.61E-04 | -4.54E-04 | 1.38E-03 | 0.08 | 2.07 | 0.151 |
| pP53_nuclei | -9.05E-04 | -3.35E-03 | 1.54E-03 | -0.05 | 0.84 | 0.361 |
| Ki67_nuclei | -2.94E-04 | -6.65E-04 | 7.79E-05 | -0.05 | 1.27 | 0.259 |
| pHH3_nuclei | 8.17E-03 | -3.15E-03 | 1.95E-02 | 0.08 | 2.73 | 0.099 |
| caspase3_nuclei | 3.03E-02 | 9.62E-03 | 5.09E-02 | -0.01 | 0.11 | 0.742 |
| pBetaCatenin_nuclei | 4.14E-03 | 1.17E-04 | 8.16E-03 | 0.00 | 0.00 | 0.954 |
| Erbeta1_nuclei | -2.44E-04 | -2.33E-03 | 1.84E-03 | 0.03 | 0.33 | 0.568 |
| Erbeta1_cytoplasmic | 1.44E-03 | -3.36E-03 | 6.25E-03 | -0.01 | 0.02 | 0.889 |
| Erbeta2_nuclei | 4.20E-03 | -2.78E-03 | 1.12E-02 | -0.01 | 0.09 | 0.764 |
| Erbeta2_cytoplasmic | -8.83E-03 | -2.00E-02 | 2.34E-03 | 0.06 | 1.93 | 0.164 |
| WT1_nuclei | -1.67E-05 | -7.16E-05 | 3.82E-05 | 0.03 | 0.38 | 0.539 |
| Ecadherin_cytoplasm | -1.06E-05 | -7.37E-05 | 5.25E-05 | 0.04 | 0.88 | 0.349 |
| Slug_cytoplasm | 7.45E-06 | -8.71E-05 | 1.02E-04 | 0.04 | 0.67 | 0.413 |
| Snail_nuclei | -3.26E-05 | -1.01E-04 | 3.59E-05 | -0.06 | 1.27 | 0.260 |
| ***Progression-free survival, LASSO feature selection*** | | | |  |  |  |
| ***Proteomics*** | | | |  |  |  |
| caspase3_nuclei | 1.11E-02 | -9.08E-03 | 3.12E-02 | -0.04 | 0.69 | 0.407 |
| pBetaCatenin_nuclei | 1.86E-03 | -7.89E-04 | 4.51E-03 | 0.03 | 0.45 | 0.504 |
| Ecadherin_cytoplasm | -3.38E-05 | -8.58E-05 | 1.82E-05 | 0.03 | 0.31 | 0.581 |
| ***Clinicopathological*** | | | |  |  |  |
| Stage | 4.32E-01 | 2.83E-01 | 5.82E-01 | 0.02 | 0.19 | 0.667 |
| Regimen=platinum+taxane | -3.45E-01 | -5.67E-01 | -1.23E-01 | 0.15 | 6.93 | 0.009 |
| ***Combined*** | | | |  |  |  |
| Stage | 4.61E-01 | 3.09E-01 | 6.12E-01 | 0.02 | 0.08 | 0.781 |
| Regimen=platinum+taxane | -3.50E-01 | -5.73E-01 | -1.28E-01 | 0.16 | 8.34 | 0.004 |
| caspase3_nuclei | 2.04E-02 | 9.72E-05 | 4.07E-02 | -0.01 | 0.11 | 0.741 |
| pBetaCatenin_nuclei | 2.26E-03 | -5.15E-04 | 5.04E-03 | 0.01 | 0.03 | 0.858 |
| Ecadherin_cytoplasm | -3.31E-05 | -8.46E-05 | 1.84E-05 | 0.05 | 0.92 | 0.338 |
| ***Overall survival, no feature selection*** | | | |  |  |  |
| ***Proteomics*** | | | |  |  |  |
| pERK_nuclei | -1.03E-04 | -1.40E-03 | 1.20E-03 | -0.01 | 0.07 | 0.797 |
| pSTAT3_727_nuclei | 1.84E-04 | -1.22E-03 | 1.59E-03 | -0.09 | 2.86 | 0.091 |
| pSTAT3_705_nuclei | -2.03E-04 | -1.58E-03 | 1.17E-03 | 0.09 | 2.44 | 0.118 |
| pNFkB_nuclei | -2.55E-03 | -5.93E-03 | 8.28E-04 | 0.04 | 0.33 | 0.563 |
| pBRCA1_nuclei | -3.28E-03 | -8.66E-03 | 2.10E-03 | -0.03 | 0.40 | 0.527 |
| pH2AX_nuclei | 8.14E-04 | -6.29E-04 | 2.26E-03 | -0.01 | 0.05 | 0.818 |
| pRB_nuclei | 4.40E-04 | -4.85E-04 | 1.37E-03 | 0.07 | 1.51 | 0.219 |
| pP53_nuclei | -1.10E-03 | -3.90E-03 | 1.70E-03 | -0.07 | 1.68 | 0.194 |
| Ki67_nuclei | -2.38E-04 | -6.06E-04 | 1.29E-04 | -0.03 | 0.42 | 0.517 |
| pHH3_nuclei | 8.55E-03 | -2.57E-03 | 1.97E-02 | 0.06 | 1.01 | 0.316 |
| caspase3_nuclei | 2.26E-02 | 1.98E-03 | 4.33E-02 | 0.00 | 0.00 | 0.990 |
| pBetaCatenin_nuclei | 2.02E-03 | -1.97E-03 | 6.01E-03 | 0.00 | 0.01 | 0.944 |
| Erbeta1_nuclei | -1.13E-03 | -3.24E-03 | 9.86E-04 | 0.00 | 0.00 | 0.967 |
| Erbeta1_cytoplasmic | 1.39E-03 | -3.33E-03 | 6.11E-03 | -0.01 | 0.04 | 0.844 |
| Erbeta2_nuclei | 7.28E-03 | 3.90E-04 | 1.42E-02 | -0.03 | 0.26 | 0.608 |
| Erbeta2_cytoplasmic | -7.97E-03 | -1.85E-02 | 2.53E-03 | 0.09 | 3.21 | 0.073 |
| WT1_nuclei | -6.26E-06 | -5.80E-05 | 4.55E-05 | 0.08 | 2.23 | 0.136 |
| Ecadherin_cytoplasm | -4.99E-05 | -1.13E-04 | 1.27E-05 | -0.09 | 3.41 | 0.065 |
| Slug_cytoplasm | 1.51E-05 | -7.61E-05 | 1.06E-04 | 0.03 | 0.29 | 0.591 |
| Snail_nuclei | 4.86E-06 | -6.62E-05 | 7.59E-05 | -0.01 | 0.03 | 0.859 |
| ***Clinicopathological*** | | | |  |  |  |
| Age | 1.39E-05 | -2.64E-05 | 5.42E-05 | 0.12 | 4.35 | 0.037 |
| AgeStratified | -1.22E-01 | -5.54E-01 | 3.10E-01 | -0.06 | 1.12 | 0.289 |
| HistologicalType=clear_cell | -3.32E-02 | -8.92E-01 | 8.25E-01 | 0.06 | 1.29 | 0.256 |
| HistologicalType=endometrioid | -5.66E-01 | -1.29E+00 | 1.52E-01 | -0.02 | 0.08 | 0.781 |
| HistologicalType=mixed_histology | -2.15E-01 | -9.69E-01 | 5.39E-01 | -0.04 | 0.56 | 0.456 |
| HistologicalType=mucinous | 3.59E-01 | -6.39E-01 | 1.36E+00 | -0.01 | 0.01 | 0.904 |
| HistologicalType=papillary_serous | -3.28E-01 | -1.01E+00 | 3.52E-01 | -0.01 | 0.04 | 0.835 |
| Stage | 4.30E-01 | 2.60E-01 | 6.00E-01 | -0.02 | 0.12 | 0.733 |
| Regimen=platinum+taxane | -2.89E-01 | -5.44E-01 | -3.36E-02 | 0.19 | 11.78 | 0.001 |
| ***Combined*** | | | |  |  |  |
| Age | 1.59E-05 | -2.79E-05 | 5.96E-05 | 0.14 | 6.55 | 0.011 |
| AgeStratified | -2.14E-01 | -6.80E-01 | 2.52E-01 | -0.10 | 3.16 | 0.075 |
| HistologicalType=clear_cell | -3.07E-01 | -1.33E+00 | 7.13E-01 | 0.08 | 2.36 | 0.125 |
| HistologicalType=endometrioid | -4.78E-01 | -1.27E+00 | 3.12E-01 | -0.01 | 0.04 | 0.838 |
| HistologicalType=mixed_histology | 2.66E-03 | -8.28E-01 | 8.33E-01 | -0.05 | 0.85 | 0.357 |
| HistologicalType=mucinous | 7.46E-01 | -3.29E-01 | 1.82E+00 | 0.03 | 0.31 | 0.578 |
| HistologicalType=papillary_serous | -9.73E-02 | -8.37E-01 | 6.43E-01 | -0.03 | 0.25 | 0.616 |
| Stage | 5.13E-01 | 3.30E-01 | 6.96E-01 | -0.05 | 0.87 | 0.352 |
| Regimen=platinum+taxane | -3.52E-01 | -6.26E-01 | -7.73E-02 | 0.19 | 13.12 | 0.0003 |
| pERK_nuclei | -5.16E-04 | -1.84E-03 | 8.03E-04 | -0.03 | 0.27 | 0.605 |
| pSTAT3_727_nuclei | 7.94E-04 | -6.87E-04 | 2.28E-03 | -0.09 | 2.90 | 0.088 |
| pSTAT3_705_nuclei | -5.21E-04 | -1.93E-03 | 8.84E-04 | 0.10 | 2.93 | 0.087 |
| pNFkB_nuclei | -3.88E-03 | -7.42E-03 | -3.39E-04 | 0.03 | 0.32 | 0.573 |
| pBRCA1_nuclei | -3.89E-03 | -9.35E-03 | 1.58E-03 | -0.03 | 0.31 | 0.580 |
| pH2AX_nuclei | 1.13E-03 | -5.67E-04 | 2.82E-03 | -0.06 | 1.01 | 0.315 |
| pRB_nuclei | 5.10E-04 | -4.72E-04 | 1.49E-03 | 0.10 | 3.31 | 0.069 |
| pP53_nuclei | -8.84E-05 | -2.80E-03 | 2.62E-03 | -0.08 | 1.85 | 0.174 |
| Ki67_nuclei | -2.74E-04 | -6.70E-04 | 1.22E-04 | -0.02 | 0.15 | 0.699 |
| pHH3_nuclei | 7.37E-03 | -3.48E-03 | 1.82E-02 | 0.06 | 0.93 | 0.334 |
| caspase3_nuclei | 3.46E-02 | 1.27E-02 | 5.64E-02 | -0.04 | 0.90 | 0.342 |
| pBetaCatenin_nuclei | 3.51E-03 | -8.22E-04 | 7.85E-03 | -0.05 | 1.00 | 0.316 |
| Erbeta1_nuclei | -8.21E-04 | -3.05E-03 | 1.40E-03 | 0.01 | 0.06 | 0.806 |
| Erbeta1_cytoplasmic | 1.98E-03 | -3.05E-03 | 7.01E-03 | -0.01 | 0.01 | 0.908 |
| Erbeta2_nuclei | 7.05E-03 | -9.99E-05 | 1.42E-02 | -0.05 | 1.09 | 0.297 |
| Erbeta2_cytoplasmic | -8.23E-03 | -1.93E-02 | 2.85E-03 | 0.12 | 5.64 | 0.018 |
| WT1_nuclei | -5.56E-05 | -1.11E-04 | 2.13E-07 | 0.07 | 1.83 | 0.177 |
| Ecadherin_cytoplasm | -4.05E-05 | -1.04E-04 | 2.29E-05 | -0.10 | 4.43 | 0.035 |
| Slug_cytoplasm | 3.55E-07 | -9.98E-05 | 1.01E-04 | 0.06 | 1.32 | 0.250 |
| Snail_nuclei | -4.63E-07 | -7.10E-05 | 7.01E-05 | -0.02 | 0.13 | 0.716 |
| ***Overall survival, LASSO feature selection*** | | | |  |  |  |
| ***Proteomics*** | | | |  |  |  |
| pH2AX_nuclei | 9.94E-04 | -4.22E-04 | 2.41E-03 | -0.02 | 0.16 | 0.693 |
| caspase3_nuclei | 2.00E-02 | 8.22E-04 | 3.92E-02 | -0.01 | 0.01 | 0.904 |
| pBetaCatenin_nuclei | 9.85E-04 | -1.93E-03 | 3.90E-03 | 0.02 | 0.13 | 0.717 |
| WT1_nuclei | -1.06E-05 | -4.93E-05 | 2.82E-05 | 0.13 | 4.53 | 0.033 |
| Ecadherin_cytoplasm | -5.10E-05 | -1.08E-04 | 5.51E-06 | -0.11 | 4.84 | 0.028 |
| ***Clinicopathological*** | | | |  |  |  |
| Stage | 4.14E-01 | 2.49E-01 | 5.79E-01 | -0.03 | 0.35 | 0.555 |
| Regimen=platinum+taxane | -3.09E-01 | -5.46E-01 | -7.23E-02 | 0.16 | 7.61 | 0.006 |
| ***Combined*** | | | |  |  |  |
| Stage | 4.82E-01 | 3.12E-01 | 6.52E-01 | -0.07 | 1.54 | 0.214 |
| Regimen=platinum+taxane | -3.41E-01 | -5.79E-01 | -1.03E-01 | 0.16 | 7.98 | 0.005 |
| pH2AX_nuclei | 1.19E-03 | -3.61E-04 | 2.74E-03 | -0.05 | 0.74 | 0.390 |
| caspase3_nuclei | 2.85E-02 | 9.41E-03 | 4.75E-02 | -0.01 | 0.02 | 0.874 |
| pBetaCatenin_nuclei | 1.87E-03 | -1.18E-03 | 4.93E-03 | 0.00 | 0.00 | 0.971 |
| WT1_nuclei | -3.97E-05 | -8.10E-05 | 1.52E-06 | 0.14 | 5.81 | 0.016 |
| Ecadherin_cytoplasm | -4.50E-05 | -1.00E-04 | 1.01E-05 | -0.11 | 4.14 | 0.042 |

## Notes

**Supplementary Note S1. R vignette of Monte Carlo analysis.**

This vignette shows an example of running the ‘MConCindex’ function to recreate the Monte Carlo (MC) results reported in Fig. 1k: evaluating the p-value of the C-index from the CHPR model using the LASSO-selected features for the proteomics and clinicopathological data combined, only shuffling the proteomics variables.

The ‘MConCindex’ function takes seven parameters; the first three are required:

- formula – a formula for the cph function of package rms
- datain – a dataframe containing the data to analyse
- shuffle – a vector of the variable names to shuffle, as character strings
- numreps – number of repetitions for the MC analysis (default = 1000)
- filename – a file name to write the values from each MC repetition (default = NULL)
- cv – set to TRUE to run 10-fold cross-validation within the MC (default = FALSE)
- cvtype – state to use the c-index values from the test sets in cross-validation (“test”) or the corrected c-index (“corrected”) (default = “test”)

The function returns six values:

- MC.c.values – c-index for each MC repetition (all cross-validation values returned if cv=TRUE)
- model.c.value – c-index for actual analysis
- num.MC.repetitions – number of MC repetitions used
- num.greater – number of MC repetitions with equal or greater c-index
- p.value – p-value of the MC test
- cross.validation – type of cross-validation (none, test, or corrected)
- formula – the formula for the cph function
- shuffled.variables – the variables shuffled in the MC analysis

The package rms is required to run this vignette. Change R’s working directory to the folder containing the Verleyen_MonteCarloCPH.R file and the supplementary data file Verleyen_Dataset.csv. Begin by loading the functions and data.

*> source("Verleyen_MonteCarloCPH.R")*

*> OvarianData <- read.csv("Verleyen_Dataset.csv")*

*> names(OvarianData)*

*[1] "Age" "AgeinYears" "AgeStratified"*

*[4] "HistologicalType" "Stage" "Regimen"*

*[7] "PFS" "Recurred" "OS"*

*[10] "Dead" "pERK_nuclei" "pSTAT3_727_nuclei"*

*[13] "pSTAT3_705_nuclei" "pNFkB_nuclei" "pBRCA1_nuclei"*

*[16] "pH2AX_nuclei" "pRB_nuclei" "pP53_nuclei"*

*[19] "Ki67_nuclei" "pHH3_nuclei" "caspase3_nuclei"*

*[22] "pBetaCatenin_nuclei" "Erbeta1_nuclei" "Erbeta1_cytoplasmic"*

*[25] "Erbeta2_nuclei" "Erbeta2_cytoplasmic" "WT1_nuclei"*

*[28] "Ecadherin_cytoplasm" "Slug_cytoplasm" "Snail_nuclei"*

Call MConCindex with this data and the feature-selected model, shuffling only the proteomics variables. Running with 10,000 repetitions, as below, can take 20-30 minutes or more depending on processor speed. To shorten the runtime, lower the repetitions.

*> LASSOBothMC <- MConCindex(Surv(PFS,Recurred) ~ Stage + Regimen + caspase3_nuclei + pBetaCatenin_nuclei + Ecadherin_cytoplasm, OvarianData, c("caspase3_nuclei","pBetaCatenin_nuclei","Ecadherin_cytoplasm"),10000)*

*[1] "loop 1"*

*[1] "loop 2"*

*[1] "loop 3"*

*[1] "loop 4"*

*[1] "loop 5"*

*...*

*[1] "loop 9995"*

*[1] "loop 9996"*

*[1] "loop 9997"*

*[1] "loop 9998"*

*[1] "loop 9999"*

*[1] "loop 10000"*

*There were 11 warnings (use warnings() to see them)*

Note that with random data, there may be occasional convergence problems in the Cox model; warnings about “Loglik converged before variable” can be safely ignored for the purposes of the MC test.

The p-value can then be retrieved. Other useful features of the analysis are also stored.

*> LASSOBothMC$p.value*

*[1] 0.0213*

*> LASSOBothMC$model.c.value*

*[1] 0.6214351*

*> LASSOBothMC$formula*

*Surv(PFS, Recurred) ~ Stage + Regimen + caspase3_nuclei + pBetaCatenin_nuclei +*

*Ecadherin_cytoplasm*

*> LASSOBothMC$shuffled.variables*

*[1] "caspase3_nuclei" "pBetaCatenin_nuclei" "Ecadherin_cytoplasm"*

## Data

**Supplementary Data S1. Proteomics and clinicopathological data.** Provided as an excel (.xls) file.

**Supplementary Data S2. R code for Monte Carlo analysis.** Provided as text (.R) file.
